# CRISPR-Cas Technology: Emerging Applications in Clinical Microbiology and Infectious Diseases

**DOI:** 10.3390/ph14111171

**Published:** 2021-11-17

**Authors:** Sahar Serajian, Ehsan Ahmadpour, Sonia M. Rodrigues Oliveira, Maria de Lourdes Pereira, Siamak Heidarzadeh

**Affiliations:** 1 Cell Science Research Center, Department of Molecular Systems Biology, Royan Institute for Stem Cell Biology and Technology, ACECR, Tehran 16635-148, Iran; haropcs@gmail.com; 2 Infectious and Tropical Diseases Research Center, Tabriz University of Medical Sciences, Tabriz 51666-14766, Iran; ehsanahmadpour@gmail.com; 3 Immunology Research Center, Tabriz University of Medical Sciences, Tabriz 51666-14766, Iran; 4 CICECO-Aveiro Institute of Materials, University of Aveiro, 3810-193 Aveiro, Portugal; sonia.oliveira@ua.pt; 5 Hunter Medical Research Institute, New Lambton, NSW 2305, Australia; 6 Department of Medical Sciences, University of Aveiro, 3810-193 Aveiro, Portugal; 7 Department of Microbiology and Virology, School of Medicine, Zanjan University of Medical Sciences, Zanjan 45139-56184, Iran

**Keywords:** CRISPR, gene editing, infectious diseases, antimicrobial resistance, parasitology

## Abstract

Through the years, many promising tools for gene editing have been developed including zinc-finger nucleases (ZFNs), transcription activator-like effector nucleases (TALENs), CRISPR-associated protein 9 (Cas9), and homing endonucleases (HEs). These novel technologies are now leading new scientific advancements and practical applications at an inimitable speed. While most work has been performed in eukaryotes, CRISPR systems also enable tools to understand and engineer bacteria. The increase in the number of multi-drug resistant strains highlights a necessity for more innovative approaches to the diagnosis and treatment of infections. CRISPR has given scientists a glimmer of hope in this area that can provide a novel tool to fight against antimicrobial resistance. This system can provide useful information about the functions of genes and aid us to find potential targets for antimicrobials. This paper discusses the emerging use of CRISPR-Cas systems in the fields of clinical microbiology and infectious diseases with a particular emphasis on future prospects.

## 1. Introduction

Over the past 60 years, extraordinary progress has been achieved in genetics and biology, especially by manipulating DNA. Exploring the double helix structure of DNA was a milestone for further advancements such as the solid-phase DNA system, genome sequencing technologies that have enabled researchers to detect and explore genome organization [1,2]. Recent advances in genetic engineering tools show promising transformation in many fields, from identifying variation in genomes to making site-specific alterations and manipulating the genome of organisms and cells in their endogenous content [1,2,3].

Finding an odd sequence of repetitive DNA in *Escherichia coli* was a strong base for later genome engineering and had a major impact on our understanding of bacterial immunology. It has been two decades since researchers discovered many organisms that contain these repeated genomic sequences with 20–58 base pair sequences between them. These elements were called clustered regularly interspaced short palindromic repeats (CRISPR) by the scientific community [4,5].

These repetitive sets were found in 90% of archaea and almost half of bacteria species, but not in eukaryotes and viruses [4,6]. Through the years, many promising tools for gene editing have been developed including zinc-finger nucleases (ZFNs), transcription activator-like effector nucleases (TALENs), CRISPR-associated protein 9 (Cas9), and homing endonucleases (HEs). These novel technologies are now leading new scientific advancements and practical applications at an inimitable speed [7]. While most work has been performed in eukaryotes, CRISPR systems also enable tools to understand and engineer bacteria [8]. The increase in the number of multi-drug-resistant strains highlights a necessity for more useful treatment options. This system can provide useful information about the functions of genes and aid us to find potential targets for antimicrobials [9]. This paper discusses CRISPR-Cas systems and other applications in microbiology and infectious diseases with a particular emphasis on historical background, future prospects, and challenges.

## 2. Overview of CRISPR Cas System

As mentioned before, a breakthrough in genome engineering was based on a study by a Japanese researcher who identified an odd sequence of repetitive DNA in *E. coli*. This sequence of repetitive DNA (spacers) was derived either from mobile genetic elements (MGEs) or bacteriophage [10].

CRISPR-associated (Cas) genes recognize these repeats. Three major elements create the CRISPR locus, which are Cas genes, spacer arrays, and leader sequences. The Cas protein with spacers of CRISPR array spots and destroys the invading DNA with the aid of protospacers [11]. Adaptation, expression, and interference are three processes that take down the foreign nucleic acids [12].

First, the adaptation stages include integration of a section of foreign DNA into its protospacer adjacent motifs (PAMs) and CRISPR array by the organism from spacer sequences of the host genome. Secondly, in the expression stage, crRNA is developed from the transcription of RNA from pre-crRNA. At last, in the interference stage, crRNA and Cas protein together recognize the foreign DNA, which result in cleaving the target and making a double-strand break, which ends the overall process.

Therefore, CRISPR/Cas9 gene targeting requires a custom single guide RNA (sgRNA) that contains a targeting sequence (crRNA sequence) and a Cas9 nuclease-recruiting sequence (tracrRNA). The sgRNA is composed of 20 nucleotides complementary to a sequence in genome flanking protospacer-adjacent motif (PAM); thus, this 20-nucleotide sequence is homologous to a region in the gene of interest and will direct Cas9 nuclease activity.

This was an overview of the CRISPR-Cas system, which was later classified into classes and various types and subtypes [10]. The description of the CRISPR/Cas9 system has been published by Lone et al. directed to bacterial genomic engineering [13].

## 3. Historical Background

In 1979, Scherer et al. established the development of gene replacement [14]. By inserting the selected set of sequences into introns, researchers hypothesized that desired genetic information within a genome could be synthesized [15]. At around the same time, some reports presented zinc finger-mediated DNA binding, which paved the way for modular DNA recognition proteins. The zinc finger nucleases (ZFNs) method was effective in changing the genomic sequence of the mammalian cell and Drosophila [16]. Some experiments were carried out using the ZFNs method; however, it did not become a widely adopted technique [1].

Another method that initially generated excitement was the transcription activator–like effector nucleases (TALENs) method. This technique had some similarities to ZFNs due to site-directed genome-editing features, and also it was simpler and cheaper than ZFNs [17]. In the mid-2000s, a few researchers were investigating a set of DNA sequences called CRISPR, which were previously discussed by Ishino et al. in 1987 and observed in other bacteria and archaea by Mojica et al. in 2000 [18,19].

**Figure 1:**
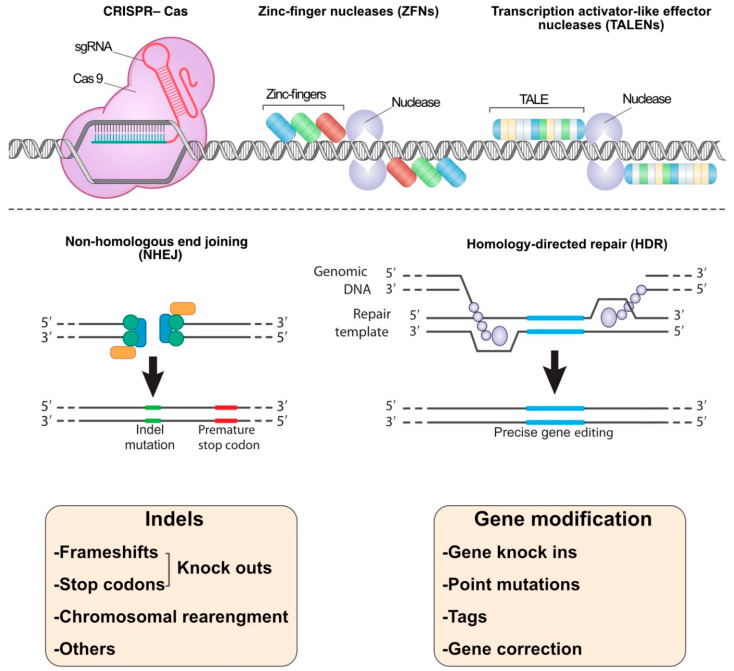
The common nucleases in the genome-editing methods: CRISPR, TALENs, and ZFNs systems. These nucleases break double-strand DNA targets, and then the breakage sites are repaired by non-homologous end joining (NHEJ) or homology-directed repair (HDR) mechanisms. Indels (refer to insertion–deletion mutations) may lead to gene knockouts and chromosomal rearrangement. HDR methodology could be used in gene modification i.e., gene knock-ins, point mutations, tags adding, and gene editing.

A key insight came through when Bolotin et al. demonstrated that CRISPR spacer sequences were originated from virus and plasmids [20]. These observations led to a hypothesis that the CRISPR-Cas system may be an adoptive defense system in bacteria that uses antisense RNAs as a trace of past invasions [21]. In 2007, Barrangou et al. demonstrated the first experimental evidence of adaptive immunity mediated by the CRISPR-Cas system on the bacterium *Streptococcus thermophilus* with lytic phages [22]. A year later, the foundation of the CRISPR-Cas system as a DNA targeting tool was discovered through the identification of CRISPR RNAs (crRNAs) as guides with Cas proteins in a complex (Figure 1) [23]. Subsequently, the classification of three major classes of the CRISPR-Cas system was pointed out by Makarova et al. [11]. The first human trial to utilize CRISPR gene editing gained approval from the National Institute of Health (NIH) advisory committee for the use in cancer therapy [24]. Researchers from the UT Southwestern Medical Center reported the first utilization of CRISPR genome-wide screening to distinguish a gene that aids cells to resist flavivirus infection [25].

CRISPR system has a wide range of applications in gene function studies, diagnostic, and therapeutics. This system is one of powerful tools in alternation and editing genes, such as DNA labeling, inducible regulation for specific control, multiplexing experiment designing, isolation of specific genome region, activation and repression to inactivate Cas9, among others, and some other applications of this system are available in Table 1 [26,27].

## 4. Applications of CRISPR System

Genomic manipulation is humankind’s hope for curing monogenic disease and, more than that, a way to treat different cancers, infections, and degenerative diseases. Previously, genetic therapies utilized technologies such as ZFNs and TALENs, but with promising technology such as the CRISPR-Cas system, the ability to edit genes become more specific and more flexible than traditional technology [44]. Furthermore, the CRISPR-Cas system offers good promise in applying this system in preclinical models of disease such as cultured human cells and animal models [44].

There are several approaches of therapeutics application of this system, but the most appropriate way will be dependent on the target tissue, the path of administration, and the type of editing [45].

As first approach, therapeutic applications are performed in a laboratory setting by removing cells from the targeted body, manipulating genes with the CRISPR-Cas system, and putting the altered cells back by transplantation into the patients [46]. Based on the tissue in need of correction, it can be either blood and/or bone marrow that should be edited. For example, in Wiskott Aldrich syndrome, the targets for the gene editing are hematopoietic stem/progenitor cells (HSPCs) [47]. In the auto-immune diseases such as rheumatoid arthritis, the target cells can be regulatory T cells. It is likely that, with the CRISPR-Cas system, this progress will accelerate. Ex vivo therapeutics heavily rely on autologous transplantation, but it is appealing if genome editing goes toward engineered “universal donor” T cells or stem cells [48].

Another approach in genome editing is adoptive cell transfer, which is a type of immunotherapy. One of these gene transfer therapies is a chimeric antigen receptor (CAR) T-cell therapy, which is the first gene transfer therapy approved by US Food and Drug Administration (FDA) [49]. In this method, T cells have a knockout of programmed cell death protein 1 (PD-1), and then they are programmed to recognize cancerous cells through an engineered extracellular antigen-binding domain. The first time CRISPR was used for the treatment of cancer was in 2016 on a metastatic non-small cell lung cancer patient at Sichuan University’s West China Hospital in Chengdu [50].

Another application of CRISPR is for treating sickle-cell disease or β-thalassemia, which are both hemoglobinopathies caused by genetic aberrations. Researchers proposed several treatment strategies, and one involved ex vivo gene editing of CD34^+^ hematopoietic cells (HSPCs). In 2019, a patient named Victoria Gray was the first attempt to treat sickle cell disease with CRISPR technology, which involved using CTX001 gene-editing stem cell therapy to edit the patient’s hematopoietic stem cells. Until now observation of her in the Sarah Cannon Research Institute in Nashville, Tennessee, has shown 94.7% of her RBC contain edited protein and 46.6% of hemoglobin in her system is still fetal hemoglobin as an outcome of a single dose of CDX001 [26].

Hopefully, the progress achieved in ex vivo CRISPR therapeutics will translate to in vivo-compatible administration of genome-editing platforms for a wide variety of diseases. Another step in this transformation is patient-derived xenografts (PDXs), which are reliable in vivo models to investigate various aspects of human cancer. Before, the limitation was the inability to perform targeted genome editing of tumors, but the development of the CRIPSR-Cas9 system enables researchers to perform rapid antibody-based selection of transduced cells without the requirement of in vitro culturing [51].

The combination of a DNA repair template and homology-directed repair (HDR) can make a desired mutation; it can also be used to identify in vivo genetic dependencies [26,51].

Additionally, the CRISPR system has shown that it has the potential to be used in Duchenne muscular dystrophy, several brain diseases, eye diseases such as congenital blindness, infections, and diseases of the liver [26].

There is a need for highly sensitive, direct detection diagnostics for the detection of specific nucleic acid sequences as biomarkers of disease or infection (e.g., viral or bacterial sequences) [52].

Scientists think CRISPR-Cas enzymes have the potential to create such a technology. Cas12a and Cas13 were discovered in 2015, and both work based on cleaving nearby single-stranded DNA [53,54].

This activity can be used for in vitro detection of specific DNA or RNA by using a purified Cas12a or Cas13 enzymes technology, named SHERLOCK (specific high-sensitivity enzymatic reporter unlocking), first by employing Cas13 enabled detection of molar levels of Zika and dengue virus RNA. The other approach in detection, DETECTR (DNA endonuclease-targeted CRISPR trans reporter), applies Cas12a to molar sensitivity for human papillomavirus [55].

CRISPR-based technology has also been used to detect disease-causing mutations in patient biopsy samples. Thus, it is too early to say, but CRISPR-based diagnostic platforms have major potential for clinical application [56]. We are going to talk about the role of detection in early recognition and treatment of infections.

## 5. Identifying Functions of Genes in Bacteria

The CRISPR/Cas9 system has been utilized for screening essential bacterial genes and identifying their virulence factors and chemical vulnerabilities. Peters et al. reported a network of whole-genome interactions in Bacillus subtilis via a CRISPR-mediated knock-down screen [57]. They suggested this approach could be used in other pathogenic bacteria, which may aid identification of new intervention strategies [57]. Tao et al. (2016) performed CRISPR-Cas9-mediated genome-wide screens on *Clostridioides difficile* and identified the members of the Wnt receptor frizzled family (FZDs), where they function as toxin B (TcdB) receptors. Toxin B is a major virulence factor in *C. difficile* infection, responsible for severe symptoms of *C. difficile* [58]. In another aspect, screening the genes in bacteria is one of the main focuses of researchers, and Rousset et al. designed a new CRISPRi platform that is suitable for most *E. coli* isolates and closely related species. Due to this technique, designing a custom sgRNA (single guide RNA) library to target a subset of specific genus is possible. With this design, more than 90% of *E. coli* genes present in sequenced isolates can be targeted and screened [59].

The ability to make mutations and analyze the effect of those was the next step. One of the reports in the assessment of gene function in bacteria belongs to Garst and his team. They offered a high-throughput method to make mutations and analyze their phonotype. They reported a novel method that combines CRISPR-Cas9 gene editing with parallel oligomer synthesis called CREATE (CRISPR-enabled trackable genome engineering), enabling trackable mutations on a genome-wide scale [60].

In 2020, Chen et al. exploited the CREATE technology and transcriptomic analysis to search antibiotic tolerance in *E. coli*. Their results showed seven new mutations that were relatable to the ribosome-targeting antibiotics such as gentamicin, thiamphenicol, and doxycycline in *E. coli*. Their outcomes represent a novel method to aid a better understanding of the mechanisms of resistance to aminoglycoside antibiotics and tetracycline antibiotics. It can also be utilized for quickly identifying resistance-related mutations [61].

Another study about the comprehension of antibiotic resistances and sensitivities belongs to M. Peters et al. Due to their report, the lack of needed genetic tools to associate genes with phenotypes is known to be one of the major obstacles. The CRISPRi method is used as a technique to block gene expression, utilizing catalytically inactive Cas9 protein (dCas9) and single guide RNAs. They established ‘Mobile-CRISPRi’ that add genomic integration and easy transfer to different bacteria by conjugation. They demonstrated the efficacy of this method in human pathogens associated with antibiotic resistance and investigated drug–gene synergies at the library scale [62].

Additionally, RNA-guided programmable nucleases from CRISPR systems make specific breaks in DNA or RNA at desired positions. In living cells, this can lead to modifications in DNA sequence or RNA transcript abundance. Another method termed base editing is a newer genome-editing approach that exploits components from CRISPR systems together with other enzymes to directly install point mutations into cellular DNA or RNA. In this approach, no double-stranded DNA breaks are generated. DNA base editors contain a catalytically disabled nuclease fused to a nucleobase deaminase enzyme. Even some other cases comprise a DNA glycosylase inhibitor. RNA base editors make analogous changes utilizing components that target RNA. The point in this method is that base editors can directly convert one base or even base pair into another, making the efficient installation of point mutations in non-dividing cells, and there would be no excess undesired editing by-products [63].

Zheng et al. used a nickase Cas9-cytidine deaminase fusion protein to direct the conversion of cytosine to thymine within prokaryotic cells, resulting in high mutagenesis frequencies in *E. coli* and *Brucella melitensis* [64].

Although early examples of in vivo base editing are very encouraging, challenges associated with delivery, off-target editing, and generating indel formation and remain an important focus of ongoing efforts [64].

Findings in this area represent a potential of this method in pathogen and host factor recognition that take part in the pathogenesis of infections. Through using this platform to identify targetable host–pathogen interactions, we can aim to find novel therapeutics for infections.

## 6. Diagnostic Use of CRISPR-Cas

The first step in the fight against bacteria is rapid and accurate diagnosis enabling early recognition and treatment of infections. An ideal test should be rapid, accurate, easy to use, and affordable. Researchers hope CRISPR-Cas biology can assist this goal. Some studies utilized CRISPR-Cas technology for the diagnoses of infections. Pardee et al. used a combination of CRISPR-Cas9 and combined nucleic acid sequence-based amplification (NASBA) to distinguish Zika virus strains in vitro and in a macaque model [65]. Müller et al. utilized a combination of optical DNA mapping and CRISPR-Cas9 to identify antibiotic resistance genes in bacteria. In this application, a gRNA–Cas9 complex was employed to bind and cleave a specific sequence of the nucleic acid of plasmids that consist of resistance genes. Then, a fluorescent dye netropsin and YOYO-1 bind to DNA independently based on AT-rich region, leading to a unique emission to each DNA fragment. This assay helped researchers to recognize plasmids that produce different antimicrobial resistance enzymes that confer resistance to antimicrobials, such as extended-spectrum beta-lactamases, carbapenemases, and New Delhi metallo-ß-lactamase (NDM)-1 [66].

In December 2019, an outbreak of beta coronavirus severe acute respiratory syndrome (SARS)-CoV-2 began in Wuhan, China. This disease referred to as COVID-19 rapidly spread to produce a global pandemic due its person-to-person transmission. Several assays utilizing quantitative RT–PCR (qRT–PCR) approaches have been developed. However, the time needed for diagnosing patients with suspected SARS-CoV-2 has been more than 24 h. 

Kellner et al. established a novel CRISPR-based diagnostic platform that utilized nucleic acid pre-amplification with CRISPR-Cas enzymology for recognition of desired DNA or RNA sequences termed specific high-sensitivity enzymatic reporter unlocking (SHERLOCK). They provided instructions for setting up SHERLOCK assays with recombinase-mediated polymerase pre-amplification of DNA or RNA and subsequent Cas13- or Cas12-mediated detection via fluorescence and colorimetric readouts. This detection method, according to the report, is ultra-sensitive and can be performed in less than 15 min [67].

## 7. Emerging Therapeutic Applications

### 7.1. Utilizing CRISPR-Cas Systems to Fight against Bacterial Resistance

Antibiotic resistance is one of the biggest public health threats of our time, which is aggravated by the lack of available antibiotics for drug-resistant infections. By 2050, it is estimated that antimicrobial resistance will lead to 10 million deaths and cost $100 trillion unless novel approaches are developed [6].

Antimicrobial resistance happens naturally over time; often, genetic alternations aid this process. What accelerates the emergence of antimicrobial resistance is often overuse or prescription of inappropriate antibiotics. Other situations, such as lack of hygiene, poor infection, and disease prevention, cause various complications that also can lead to antibiotic resistance. Many mechanisms can develop antimicrobial resistance such as antibiotic efflux, modification of a drug target, alteration, inactivation of a drug, and even limiting uptake of a drug [10].

In antibiotic efflux, bacteria decrease the concentration of antibiotic in a cell by pumping toxic compounds out and regulating its internal environment [68]. Through the modification of a drug target mechanism, bacteria change their target site so that the drug binds poorly or not at all [69]. Often, these alterations can be brought about by point mutations in the gene. Another mechanism is drug inactivation or modification, which is developed by alternation of antimicrobials with the aid of three main enzymes such as chloramphenicol acetyl-transferases, ß-lactamases, and aminoglycoside-modifying enzymes [70], limiting uptake of a drug; this takes place when bacteria alter their cell membrane porin channel in a way that reduces permeability [71]. Therefore, mutations are one of the main reasons for antimicrobial resistance [71].

Several alternative approaches to fight antibiotic-resistant bacteria are hypothesized such as bioengineered synthetic peptides, engineered bacteriophages, and nano-antibiotics (synthesized virus-like nanoparticles), using eubiotics as growth promoters; however, there is a long way until the efficacy and accuracy of these methods can be tested [72]. CRISPR-Cas9 developments count as a revolution in gene edition and modulation. Harnessing this ability to construct these accurate scissions in target genes in drug-resistant bacteria may inform this process. The superiority of this alternative approach to others is the accuracy of the technique. Hence, a matching sgRNA (single-guide RNA) to a specific target gene can be programmed, which will provide the system capability to selectively kill bacteria [72].

One of the first models of this system was *E. coli* with an engineered type I CRISPR-Cas9 system, through which the genome was damaged with the 3′-to-5′ exonuclease Cas-3. Similar experiments with a type II CRISPR-Cas system have been performed. Like in 2014, the CRISPR-Cas9 system was used against antibiotic-resistant strains for the first time [73,74], demonstrating that an engineered CRISPR-Cas9 system can induce cell death or plasmid loss through the detection of genetic sequence associated with antibiotic resistance or virulence. Citorik et al. introduced the type II CRISPR-Cas system of *S. pyogenes* as an effective tool for programming various types of microorganisms [74]. Investigations demonstrated that type I CRISPR-Cas systems are more efficient at inducing DNA damage and cell killing, which may be either due to the large-scale DNA or as a result of this system’s exonuclease activity. While many bacteria contain this system, this method leads to incomplete death of bacterial population, which may be due to inefficient or defective delivery of the CRISPR-Cas system in the bacteria [7]. 

While one of the main concerns with the use of broad-spectrum antimicrobials is the elimination of natural microflora, CRISPR RNAs (crRNA) ensure the specificity of the system. CrRNA, with the help of tracrRNA (trans-activating crRNA), aids Cas9 endonucleases to create cleavage in the target genome sequence. This approach can form a double strand break via modifying the spacer of the CRISPR locus. However, the only restriction is the necessity of NGG motif at 3′ of the sequence of interest. All the constructing structure can be loaded into the bacteriophage, plasmid, or phagemids as a selective-lethal device for genome destruction. In bacteria, many modifications help bacteria resist antimicrobials, for example, antibiotic-modifying enzymes, such as *β*-lactamases, and conversion of host proteins [74]. Citorik et al. exerted two horizontal DNA transfer systems in which the genetic element is delivered to host bacteria: first, plasmid conjugation and, second, viral transduction [74]. The first system was convenient due to its broad host ranges and no requisite for recipient factor in DNA uptake, but the necessity of cell-to-cell contact was one of its downsides. After assessments of plasmid conjugation, Citroik et al. presented its low conjugation efficiency and limited RGN efficacy. However, the second mechanism in which a M13 phagemid vector was used was more promising. Target genes in this experiment were the blaSHV-18 or blaNDM-1, which granted pan-resistance to a broad spectrum and β-lactam antibiotics.

Applying phagemid of *E. coli* with resistant genes as a cargo resulted in a significant reduction in the cell of interest. This process of cell death, which was followed by cleavage of endogenous plasmid, originated from plasmid-borne toxin–antitoxin system activation. In the Citorik et al. experiment, only strains with the gyrAD87G mutation were killed by the targeted phagemid and not others, indicating the specificity of the target [74]. To test the adaptability of the CRISPR-Cas9 system, Citroik et al. designed a structure to combat the eae gene (the product of this gene is a surface adhesin in *E. coli* O157:H7 (EHEC), which aids bacteria to colonize in the intestine and damage tissue). However, the decline in *E. coli* O157:H7 (EHEC) was not significant, which might have been due to defective delivery. Their experiment represented the feasibility of utilizing this system in fighting against bacteria [74].

Bikard et al. used ΦNM1 phage encoded with CRISPR-Cas9 to detect and target resistance in virulent strains of *S. aureus*, targeting methicillin-resistance gene *mecA*, leading to a reduction in *mecA*-carrying *S. aureus* in mixed cultures. However, targeting plasmids that were comprised of tetracycline resistance in bacteria did not lead to cell killing. They combined spacers targeting antibiotic resistance with a particular spacer that gives antibiotic-sensitive bacteria the advantage of protection from phages that lysogenize bacteria. Interestingly, in this system, immunization against the transfer of an antimicrobial-resistant plasmid in non-pathogenic strains can happen. Some experiments in vivo were carried out with a mouse model with a skin infection. They compared the colonization of bacteria before and after treating the model with a phage-encoded CRISPR-Cas9 system, indicating a considerable decrease in bacterial colonization [73].

While the CRISPR-Cas system provided new ways through which bacteria can be re-sensitized to antibiotics, these bacteria have no selective benefit over antibiotic-resistant bacteria, therefore leading to the conservation of residual resistant bacteria in the population [73,75]. To solve this problem, Yosef et al. developed a technology utilizing a temperate and lytic phage for re-sensitizing bacteria to β-lactam antibiotics [76]. In this study, a temperate phage delivered the CRISPR-Cas system to target AMR genes (antimicrobial resistance), which also conferred resistance to the lytic phage. Through this novel approach, these bacteria had a selective advantage against others [76]. This represents opportunities for preservation of antibiotic-sensitive strains or even lets commensal bacteria survive and occupy the niches. One of the barriers in exploiting this method is its complexity in transferring phagemid to an environment more intricate than mouse skin, which requires more investigation. While phagemids are beneficial in experiments, they are inadequate due to lack of wild host range, the requirement of large-amount production, and their purity. These numerous problems needs to be explored further [75].

### 7.2. Driving Bacterial Gene Expression (dCas9)

Regulation of gene transcription is another application of the CRISPR-Cas system. By the help of deactivated (catalytically dead) Cas9 enzyme (dCas9), which has preserved its capability to identify and bind to a target DNA sequence, scientists can down-regulate or up-regulate gene transcription. When dCas9 is utilized for gene repression, this is called CRISPRi (CRISPR interference), and when it is used for activation of genes, this is termed CRISPRa (CRISPR activation) [7,28]. In this application, instead of typical DNA cleavage, the dCas9 enzyme is maintained in the target DNA sequence and can disrupt RNA polymerase or transcription factor binding [75,77]. Gilbert et al. used these tools to screen sensitivity to a cholera–diphtheria toxin [78]. 

### 7.3. Delivering Antibacterial to Intracellular Bacterial Infections

While the delivery of a specific sequence to bacteria is possible through the mediation of phages or several other vectors using the CRISPR-Cas9 system, the delivery becomes more challenging in intracellular pathogens. Therefore, the phage encoding the CRISPR-Cas9 system must not only selectively deliver cargo to the residing pathogen but also initially pass the host cell barrier. Although this procedure is complex, the presence of two layers increases the specificity of delivery. The delivery becomes more challenging with phage structure diversity and elimination of nanoparticle delivery. Different strategies were explored, including liposomal encapsulation, such as in the Carnes et al. [79] experiment, which employed evaporation induced a self-assembly procedure to encapsulate the bacterial element into silica- and lipid-based particle construction. Different biological components such as stabilizing protein or the presence of silica can aid modification of cargoes; for example, silica interacts with the polymer or lipid layer and mask particle to escape the immune system [9].

One of the major challenges of applying the CRISPR-Cas system to antimicrobials is the development of vectors that can transfer exogenous DNA into the specific bacteria. Delivery of the CRISPR-Cas system can happen through various techniques. Polymer-derivatized CRISPR nanoparticles, conjugative plasmids, and phages are some of techniques [80].

Phages are predators of bacteria, bind to receptors at the bacteria surface, and inject their genome into the cytoplasm. Two types of phage vector have been used with the CRISPR-Cas system. One phagemid engineered temperate/virulent phages. The project to use phagemids in delivering an engineered CRISPR-Cas9 system into different bacterial models, such as *E. coli* or *S. aureus,* has been successful, and selective killing was observed [73,74]. However, the main limitation in utilizing phagemids is that they need helper phages to produce the complete viral vector assembly. The other kind of phages, termed virulent or temperate phages, were utilized due to their improved bactericidal properties compared other phages [81,82].

One of the major concerns in engineering phages is impairing viral assembly and replication, which, with the introduction of a large DNA fragment such as a CRISPR-Cas system into a phage, may impair delivery and genome packaging [82]. Additionally, research has shown that repurposed CRISPR-Cas systems can be efficient as antimicrobials in in vitro studies but their clinical relevance must be determining through appropriate trials. Some of the questions concerning phages still remain such as investigation of the interaction between phages and mammalian organisms (e.g., immune system) or the probability of a higher bacterial evolution rate due to the use of phage therapy. However, through the last decade, due to further investigations, optimism has risen [83].

### 7.4. CRISPR and Its Application in Parasitology

According to a WHO report, parasite infections remain one of the major causes of morbidity worldwide [84]. The lack of a vaccine, poor efficiency of drugs, and antimicrobial resistance emphasizes the need for new treatment options based on parasite biology in which new tools such as CRISPR may have a role [85,86]. 

Over the past decades, there have been extraordinary efforts to sequence more than 150 parasites’ genomes [87]. While genome sequencing helps to dissect the genome and identify its function, by introducing CRISPR, scientists are now able to manipulate genes, create new sequences, and introduce them to the genome, such as the work done on the schistosome genome [88,89]. Recently, gene-editing platforms have emerged as a treatment for parasitic diseases, which can modify the host genes required by the parasite or target the parasitic genes needed for replication [90,91,92]. This system uses Cas9 endonuclease to create a double-strand break (DSB) at a selected locus in the genome. The act of binding Cas9 to a single-guide RNA (sgRNA) achieves specificity. The DSB will then be repaired in one of these three pathways: (1) through homology-directed repair (HDR) using a provided repair template, (2) through error-prone microhomology-mediated end joining (MMEJ), or (3) through nonhomologous end joining (NHEJ). CRISPR/Cas9 facilitates deletion, insertion, or mutation of DNA with little to no genetic scarring [92].

This technique was employed to manipulate the genomes of parasites associated with high mortality such as *Plasmodium* spp., *Toxoplasma gondii*, and *Cryptosporidium* spp. While ZFN has been used widely in gene editing on *Plasmodium falciparum*, targeting capability, high cost, difficult design, and application were some of the limitations to employing this technology [93]. In 2014, Ghorbal and Wagner published the adaption of CRISPR/Cas9 technique, which enabled the manipulation of *P. falciparum* by applying different approaches to express sgRNA [94,95]. Similarly, by employing CRISPR/Cas9, they were able to efficiently generate gene knockouts in *Toxoplasma* strains [96,97,98]. *Cryptosporidium* spp. is another Apicomplexa, which can cause life-threatening chronic infection in immunosuppressed adults and people living with HIV [99]. However, limited tractability of the parasite hindered our understanding of the parasitic biology and development of new treatments. These include the dearth of molecular genetic tools, lack of continuous culture, and the absence of suitable animal models [100]. Besides these limitations in culturing parasites, genomic manipulation by conventional methods and transient transfection is less efficient. CRISPR/Cas9 technology, on the contrary, has proven itself again to overcome these restrictions. For the first time, Vinayak et al. developed a new method to isolate genetically stable parasites by transfecting *Cryptosporidium parvum* sporozoites [101]. This achievement allows us to generate the first knocked out *Cryptosporidium* generation, leading to a new avenue in culturing and understanding the biology of parasites and exploring new treatments. 

**Figure 2:**
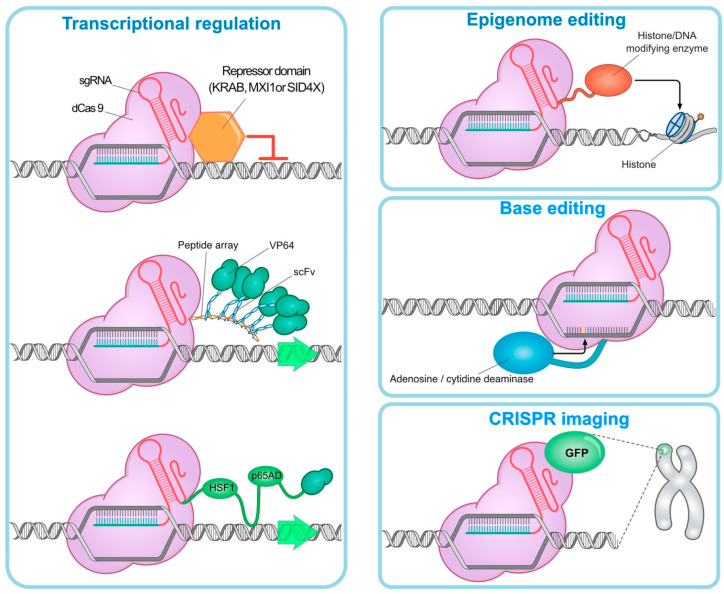
**CRISPR technology is used for the following targets: Transcription regulation:** The deactivated Cas9 (dCas9) inhibits the target gene by binding to the repressor domains (KRAB, MXI1, and SID4X) with the aid of multiple copies of the VP64 domain via a multimeric peptide array activation of endogenous genes progressed. **Epigenomic editing:** dCas9 can lead to transcriptional suppression or activation by binding to epigenetic regulators. **Base editing:** Fusion of dCas9 with adenosine deaminase (by converting adenosine to inosine) or cytidine deaminase (by converting cytidine to uridine) can be used to create single base pair edits without DSBs. **CRISPR imaging:** dCas9 can dynamically detect specific DNA in viable cells by binding to fluorescent molecules.

Similarly, *Strongyloides stercoralis* and its motility gene SS-unc-22 was knocked out through CRISPR/Cas9, resulting in severe motility defects [102]. Janssen et al. altered genes of *Trichomonas vaginalis*, one of the most common sexually transmitted infections affecting more than 250 million people around the world, by using CRISPR/Cas9 technology. In this study, they knocked out two endogenous genes by replacing a drug resistance gene cassette [103]. *Trypanosoma brucei* was benefited from genetic tool developments prior to other parasites due to efficient homologous recombination, relatively high transfection efficiencies, inducible systems such as RNAi, the tetracycline repressor, and the ability to knock out and knock in genes [104]. Recent advances in CRISPR/Cas9 technology in *T. brucei* have impressively progressed the gene-editing toolbox, leading to quick gene tagging and gene knockout and accurate editing in life cycle forms of the parasite [105] (Figure 2). Hence, CRISPR/Cas9 has become straightforward, cost-effective, and increasingly capable of introducing point mutations, creating gene knockouts, tagging endogenous genes, altering the epigenetic landscape, and changing gene expression. With the continuous and increasing progress in the use of CRISPR-Cas9 system, it has become possible to create accurate gene edits without causing higher off-target effects. Nevertheless, the absence of some pathways, such as the non-homologous end joining pathway in many parasites, limits the use of CRISPR for genome-wide screening. On other hand, better understanding of repair pathways such as DNA double-strand break repair in parasites may help exploit alternative pathways for extensive genome functional studies [2,90,91,106].

Several other microbes have been targeted successfully by CRISPR methods (Table 2, [107]). However, the different challenges of application CRISPR technology to microbe transmission or control, resistance, off-target editing, and mutations still challenge its applicability in the field against emerging resistant pathogens, as discussed by Shabbir et al. (2019) [108].

The CRISPR/Cas9 system is actually an adaptative immune system that confers resistance to microbes by targeting the nucleic acid of invading bacteriophages in a sequence-specific manner [150]. In vivo, this has been shown, for example, to inhibit the Marek’s disease virus in transgenic chicken [110]. If, by one hand, emergence of Cas9 evasion strategies by compensatory mechanisms from eukaryotic viruses such as MDV in transgenic chickens expressing Cas9 genes is unlikely, some viruses can limit RNAi efficacy in eukaryotic cells by evolving viral suppressors of RNA; CRISPR/Cas methods can target the viral gene prior to the transcription of an mRNA, thus being more effective to inhibit the replication in vivo [151].

### 7.5. CRISPR Delivery Strategies

Multiple cargos and delivery systems have been described for CRISPR/Cas9, which include physical delivery methods such as microinjection or electroporation, viral delivery via adeno-associated virus (AAV), full-sized adenovirus or lentovirus, and non-viral delivery methods using liposomes, nanoparticles, or polyplexes. These strategies have been thoroughly reviewed by researchers such as Lino et al. [152].

An investigated in vivo delivery method is hydrodynamic delivery, which involves rapidly pushing a large volume (8–10% body weight) solution containing gene-editing cargo into the bloodstream. This method significantly affects the liver and also kidney, lung, muscle, and heart cells. This method is only used for in vivo applications, as it relies on temporarily increasing the pressure in a closed system and forcing cargo through endothelial and parenchymal cells barriers, otherwise impermeable barriers [152,153].

While in vitro and ex vivo methods have been shown to be quite successful at delivering the cargo and achieve the desired target cells and results (e.g., iTOP method), this cannot yet be transposed to clinical settings [152]. Viral vectors, for example, have been used in vitro, ex vivo and in in vivo delivery systems, but with undesired effects such as mutagenesis, limited cloning capacity, and/or triggering immune reactions. On the other hand, chemical delivery systems require extensive, time-consuming optimizations to improve their efficiency for in vivo gene editing. Thus far, nanoparticles such as of lipid or gold and extracellular vesicle-based systems (such as exosome-based) are the most promising delivery agents for CRISPRCas9 components with low toxicity and no triggering of immune response [154,155,156].

However, the in vivo applications developed so far present challenges, as, in the clinical setting, they can be traumatic and cause physiological complications as well as hepatotoxicity and have very low efficiency rates. In 2016, the first CRISPR/Cas9 clinical trial was conducted [157], registering interesting prospects for nano-delivery systems for clinical gene editing to treat and/or correct genetic diseases. Nonetheless, achieving a safe, consistent, and efficient in vivo delivery system for CRISPR technology remains a challenge, despite multiple advances.

## 8. Challenges in This Field

### 8.1. The Problem with Microbial Communities

CRISPR-Cas is an emerging method to kill or even re-sensitize resistant bacteria; however, this approach has not yet been assessed in human microbial communities consisting of billion cells per gram of matrix and thousands of species. There are some difficulties in identifying different plasmids and even mobile genetic elements containing diverse resistance genes within a single species, so it would be much more complicated to assess the real-world environment. Another major challenge in using CRISPR-Cas-based antimicrobials is the prediction of community-wide responses to perturbations. It may cause unwanted knock-in effects, such as the growth of some population by the elimination of particular plasmid, and cause a domino effect that may lead to pathogenic species outgrowth [158,159]. For example, it is known that stress-induced changes to a microbial community composition and metabolite levels are associated with increased susceptibility to *C. difficile* infection in the gut, and this shift can be linked to disorders such as diabetes [160,161]. CRISPR/Cas engineering is highly specific, allowing the accurate selection between highly similar strains in pure or mixed cultures. This selectivity and programmability of microbial removal is virtually impossible with traditional antibiotics, bacteriophages, selectable markers, or other control methods.

However, delivery and off-target editing still remains a challenge, hampering the development of “smart” antibiotics against multi-resistant microbes. Additionally, the consequences of the removal of microbial communities by CRISPR/Cas remain unknown.

### 8.2. Delivery of CRISPR-Cas System

Another challenge for the application of CRISPR-Cas systems is delivery vehicles and how to pass barriers. While resistance genes are spread in a varied range of bacteria species, they are encoded in diverse places. Additionally, phages are powerful vectors, but the host ranges of phages are limited, which is a great challenge in the delivery of CRISPR-Cas systems. Another vector for delivery is conjugative plasmids, which can be transferred between bacteria, but there are some limitations to this delivery tool, such as limited host range and conjugation efficiency and difficulty in plasmid uptake. There are many studies introducing new vectors for delivery such as silica- and lipid-based particle construction, the efficacy of which are yet to be determined [158,162].

### 8.3. Resistance against CRISPR-Cas

Another concern is the development of resistance to CRISPR-Cas. It is known that in CRISPR–phage interactions, acquiring point mutations in the sequence targeted by CRISPR-Cas is possible, which can also happen in resistant genes that are aimed for removal. Moreover, resistance could happen through CRISPR-Cas loci inactivation. Mutations in *cas* genes that are important for deletion or cleavage of target spacers can lead to resistance against CRISPR-Cas. Studies have shown that the delivery of defective CRISPR systems is more likely to happen than mutations of target sequences [163]. Evolution of resistance can also happen by a selection of anti-CRISPR (*acr*) genes. These genes are responsible for encoding small proteins that bind and inactivate essential components of the CRISPR-Cas immune system. More than 20 families of *acr* genes have been identified, targeting both type I and II CRISPR-Cas systems [164]. Evolutionary risks and consequences of CRISPR-Cas targeting resistance genes need further evaluation [158]. Therefore, off-target editing events remain a primary biosafety concern for the clinical application of CRISPR technology. The off target editing events can be influenced by gRNA sequence specify and structure, the location of mismatches in gRNA, and gRNA and Cas9 concentration. Moreover, it has been shown that mismatches in the gRNA are less tolerated in vivo than in vitro [165]. Other issues may include insertions/deletions in the vicinity of Cas9 cleavage sites, and the underlying mechanisms remain unclear. Therefore, therapeutic risks must be also carefully considered when testing this method as treatment or prevention against parasite infections [166].

### 8.4. Legislation of CRISPR-Cas-Based Antimicrobials

While the development of the CRISPR-Cas system is a novel and potential way to fight against bacteria and antimicrobial resistance genes, there are a number of social and legislative issues. Liang et al. used the CRISPR/Cas9 system to cleave the endogenous β-globin gene (HBB) (which encodes a subunit of the adult hemoglobin and is mutated in β-thalassemia). In their report, off-target cleavages and unwanted mutations in human early embryos present an obstacle to using this gene therapy technology [130].

This year, 2021, Nuñez et al. from UC San Francisco reported a modified CRISPR method to extend beyond the genome and apply to the epigenome in order to control where genes are switched on or off [145]. Furthermore, this group also showed that once the gene is switched off, it remains inert in the cell’s descendants for hundreds of generations, unless switched back on with a complementary method they called CRISPRon. Their work may pave the way for various and very important epigenetic therapies, as the epigenome is central in a range of diseases from cancer to viral infections. As the technology does not involve DNA edits, the authors claim it to be safer than conventional CRISPR therapeutics, avoiding unwanted or potentially harmful changes to the genome. Despite these authors being focused on using CRISPR to treat disease, this technology can also be adapted to less ethical applications. Such was the case back in 2018 in China.

In November 2018, Jiankui, the head of rthe esearch team at the Southern University of Science and Technology (SUST) in Shenzhen, China, announced the birth of two babies by employing the CRISPR/Cas9 genome-editing technique. In this study, chemokine receptor (CCR5) genes of embryos were knocked down by CRISPR, and a HIV-negative mother in a serodiscordant couple was impregnated with these embryos that are resistant to HIV infection. Criticisms from the public and scientific community are still ongoing. Additionally, there is an ongoing debate on the consequences of this receptor knockdown, as the function is yet unknown and neurological deficits are highly possible [167]. Another important factor that led to the ethical controversy over this study is that the mother could have taken therapeutic drugs during pregnancy to prevent the unborn children from being infected with HIV, and there was no need for gene editing. This study is the evidence of the substantial potential impact of applying a genome-editing technique to human life (see Table 2 for major landmarks in the discovery and use of CRISPR/Cas technology).

One of these legislative issues is that, as Senator Chang said, “The technology is moving faster than regulations, so it’s important to be proactive about preventing safety mishaps by amateur users of CRISPR kits. While I’m a huge proponent of supporting scientific curiosity and imagination, I’m very concerned about the amateur use of this technology and its impact on consumer and public safety”.

Sales of DIY CRISPR Kits have grown among so-called biohackers, who want to change themselves with the technology. Through these news, the United States Food and Drug Administration has stated that the sale of self-administrative gene therapy products is against the law [168].

CRISPR has already been shown to help patients suffering from blood disorders sickle cell disease and beta thalassemia, and it harbors novel potential to treat rare diseases and epigenetic-based disorders, such as against cancer and to restore vision to people blinded by a rare genetic disorder. However, countries worldwide have been relying significantly on researchers’ ethics and not producing legislation when it comes to the use of these technology for other ends such as embryonic editing and human DNA manipulation.

Only one year after the first CRISPR’s babies’ announcement by He Jiankui at the second international summit on genome editing in Hong Kong, stricter regulations were put in place in a cooperation move between the People’s Republic of China, the World Health Organization (WHO), and the Russian Federation. Today, there is even a global gene editing regulation tracker, a public resource generated by the Genetic Literacy Project (https://crispr-gene-editing-regs-tracker.geneticliteracyproject.org/ accessed on 21 October 2021), where we can see how much the world’s countries improved their legislation regarding genetic modification. However, this needs to keep being built and updated as novel methods, systems, and risks are constantly reported.

Assessing the risks in utilizing gene-editing systems in the environment as well as updating guidelines on the use of these technologies is essential. It also requires national and international communities to develop and keep updating clear legislation and guidance for this technology [158]. Together, bioethics and legal law as well as the scientific community should work together in order to assure some principles that aim to protect human dignity, safeguard the integrity of patients, and safeguard their genetic information and cells to avoid inappropriate use [169].

### 8.5. Biosafety in CRISPR-Cas

Safety often relates to protection of humans, plants, animals, and the environment from unintentional harm. The term safety was highlighted early in the use of genome-editing tools as a critical limitation that needs to be resolved before any application to humans or release into the environment. Recent examples of application of CRISPR-based tools involve treating HIV, immune cell modifications to treat cancer, or the treatment of heritable diseases. Biosafety risks in this regard include the number of off-target changes, mosaicism, and potential epigenetic effects.

There are several recommendations that can be applied to avoid the utilization of safety weaknesses in genome editing in the near future such as:

1. Raising the effort to reduce mosaicism and off-target effects through further research.
2. Utilizing safe virus systems or other less-risky vector systems to transfer genome-editing tools.
3. Working on reversal gene drives in parallel to the experiment to be able to undo the effects of gene drives.
4. Developing proportional biosafety risk classification and execution of adequate containment measures in biosafety-sensitive genome-editing experiments.

Additionally, in the governance level, providing international guidance or updating existing guidance documents on biosafety and biosecurity to cover can be helpful. The application of genome-editing technology agriculture for breeding purposes in plants and animals make new challenges to biosafety, which needs more supervision [170].

## 9. Conclusions

Research on the genetic manipulation of bacteria species has been ongoing for decades. ZFNs, TALENs, and CRISPR-Cas have been applied in many fields, and this article discussed their role in infectious diseases. CRISPR-Cas for lethal-self targeting, selective removal of targeted bacteria strains, or targeting antibiotic resistance and virulence genes may be a future solution for antimicrobial resistance. Furthermore, nuclease-deactivated Cas9 paves a new way to interfere with bacterial gene expression. However, many challenges remain, such as the safety issues, increase in escape mutants, the risk of off-target mutations, and the inefficiency of delivery methods. Despite the challenges, there is a possibility that this technology could be beneficial in infectious diseases. More investigation is required to reach a safe state to employ this technology as a therapeutic strategy and translate these preclinical studies into clinical benefit. The journey from an unknown prokaryotic immune defense system to a powerful gene-editing platform emphasizes the need for ongoing investment in research to develop new solutions to unexpected challenges.

An outbreak of (SARS)-CoV-2 and producing a global pandemic were examples of these unexpected challenges. Recently, researchers have proposed a coronavirus rapid detection method based on the CRISPR/Cas system that gives nations hope of overcoming this situation.

## Figures and Tables

**Table 1:** Applications of CRISPR system.

**Gene Function**	**Function**		**Refs**
	Repress coding/noncoding genes with CRISPR/dCas9		[28,29]
	Epigenetic Regulation		
	-DNA methyltransferase DNMT3A increased CpG methylation		[30]
	-KRAB increased the H3K9me3 mark		[31]
	Large-scale functional genomic studies		[29]
**Diagnostic**	**Method (CRISPR-based)**	**Target**	**Refs**
	SHERLOCK	ZIKA	[32]
	DETECTR	Human papillomavirus (HPV)	[33]
	PC REPORTER	Tuberculosis	[34]
	RCH	MiRNAs	[35]
	CRISPR-EXPAR	Listeriosis	[36]
**Therapeutics**	**Diseases**	**Target gene/Sequence**	**Refs**
	Cystic fibrosis	CFTR	[37]
	Human immunodeficiency virus (HIV-1)	LTR loci of integrated viral genome	[38,39]
	Duchenne muscular dystrophy (DMD)	Exon 45 of dystrophin gene	[40]
	Sickle cell anemia	β-globin (HBB)	[41,42]
	Cardiovascular disease	Pcsk9	[43]

**Table 2:** A brief history of major events in the field of CRISPR method.

Year	Major Events in the Field of CRISPR Method	Refs
**1987**	Short direct repeats were first reported in the genome of the bacterium *E. coli.*	[18]
**2000**	Repetitive sequence that Ishino found in *E. coli* also was identified in other bacteria and archaea.	[19]
**2002**	-Proposal of CRISPR name and identification of *cas* gene.	[109,110]
	-Discovery of transcript of CRISPR.	
**2003**	The first reports of experimental identification of a protein associated with CRISPR DNA repeats.	[111]
**2005**	-Discovery of source of spacers from viral origin and plasmid.	[20]
	-Proposal of idea in which CRISPR-Cas may be an adaptive defense system in bacteria.	[112]
	-Identification of protospacer-adjacent motif (PAM).	[113]
**2007**	First experimental evidence for CRISPR adaptive immune system *S. thermophilus.*	[22]
**2008**	-Identification of mature CRISPRRNAs (crRNAs) as guides with Cas proteins complex as anti-phage defense system in *E. coli*.	[23]
	-Studying the interference activity of Type III (Csm) CRISPR-Cas in *Staphylococcus epidermidis*.	[114]
**2009**	-Investigating the antiviral activity of *Pyrococcus furiosus* by CRISPR-Cas systems.	[115]
	-Identification of type III-B Cmr complex that cleaves ssRNA.	[116]
**2010**	Identification of cleavage produced by the CRISPR/Cas bacterial immune system at three nucleotides ahead of the PAM sequence.	[117]
**2011**	-Classification of three major classes of CRISPR-Cas systems: types I, II, and III.	[11]
	-Discovery of trans-activating CRISPR RNA (tracrRNA).	[118]
	-Applying the *S. thermophilus* type II CRISPR-Cas system in *E. coli*, reporting that the system is active in some distantly related organisms.	[119]
	-Discovery of “seed” sequence (the seed sequence or seed region is a conserved heptametrical sequence, which is mostly situated at positions 2–7 from the miRNA 5’-end).	[120]
**2012**	-Adaptation of type II CRISPR system (originated from *S. pyogenes*) for gene editing in mammalian cells.	[121]
	-First demonstration of programming CRISPR for targeted DNA cleavage in vitro.	[122]
**2013**	-Using Cas9 successfully for genome editing in eukaryotic cell.	[123]
	-Identification of the role of III-B system in transcription-dependent DNA interference.	[124,125]
	-First use of CRISPR-Cas system in plants.	[126]
**2014**	Crystal structure of apo-Cas9, Cas, guide RNA, and target RNA.	[127,128]
**2015**	-Crystal structure of chimeric Cmr complex.	[129]
	-CRISPR/Cas9 was utilized in human embryos. Researchers applied system to repair the HBB locus, which is responsible for β-thalassemia blood disorders when it is mutant. The experiment was not effective due to its off-target activities and impossibility of prediction of gene-editing outcomes through pre-implantation genetic diagnosis (PGD”)	[130]
**2016**	-Cmr- and Csm-mediated RNA-activated DNA cleavage discovered	[24]
	-The first human trial to apply CRISPR gene editing obtained approval from the NIH.	[131]
	-New approach to genome editing that requires no dsDNA cleavage or a donor template.	[24,132,133,134]
**2017**	-Identification of a specific CRISPR protein that targets RNA rather than DNA.	[32]
	-Developing an efficient version of the CRISPR-Cas9 system called CRISPR-Gold technology that utilizes gold nanoparticles to deliver the CRISPR/Cas9 gene-editing system to cells.	[135]
	-Identification of base editing.	[63]
**2018**	Detected pre-existing antibodies that target Cas9 proteins. Represented the possibility of immune systems responses undermining the use of CRISPR-Cas9 for gene therapy.	[136]
**2019**	-Cas12a orthologs showed-editing capacity in human cells.	[137]
	-BhCas12b was also engineered as a powerful gene-editing tool.	[138]
	-Many new subtypes of Type-V CRISPR system were identified.	[139]
	-Cas12k was found as an RNA-guided site-specific integration system in *E. coli*.	[140]
	-The Class-I CRISPR system with multiple effectors has been harnessed or using fused FokI domain.	[141,142]
**2020**	-CRISPR-Cas12-based detection of SARS-CoV-2.	[143]
	-Discovery of protein inhibitors of CRISPR-Cas systems, called anti-CRISPR (Acr) proteins.	[144]
**2021**	-‘CRISPRoff’ CRISPR-based tool to switch off genes in human cells without making a single edit to the genetic code is described.	[145]
	-FDA approves first trial investigating CRISPR gene editing as HIV cure.	[146]
	-CRISPR is used for molecular genetic control of insect vectors of virus diseases (sterile insect technique).	[147]
	-CRISPR enzyme’s ancestors reported in microbes	[148,149]

## Data Availability

The data presented in this study are available on request from the corresponding author. Authors can confirm that all relevant data are included in the article.
